# Expression of the P2X1 receptor remains in the type II spiral ganglion neurons in the mature rat cochlea

**DOI:** 10.1007/s11302-026-10129-7

**Published:** 2026-01-24

**Authors:** Prakansha N. Kumar, Srdjan M. Vlajkovic, Peter R. Thorne, Haruna Suzuki-Kerr

**Affiliations:** 1 Department of Audiology, The University of Auckland, Auckland, New Zealand; 2 Department of Physiology, The University of Auckland, Faculty of Medical and Health Sciences, 85 Park Road, Grafton, Auckland, 1023 New Zealand; 3 Eisdell Moore Centre, The University of Auckland, Auckland, New Zealand

**Keywords:** Cochlea, Adult rat, Spiral ganglion neurons, P2X1 receptor, Immunohistochemistry, Auditory neurotransmission

## Abstract

Our sense of hearing commences in the cochlea, the peripheral sensory organ for hearing. Spiral ganglion neurons (SGN) in the cochlea are primary auditory neurons responsible for auditory neurotransmission. There are two classes of SGNs: type I SGNs, which make up 90-95% of the SGN population, and type II SGNs, which make up the remainder. Previous studies have shown that SGNs express a combination of purinergic (P2X, P2Y and adenosine) receptors at the mRNA and protein levels. In this study, we have focused on the P2X1 receptor to characterise its expression pattern in the Wistar rat cochlea at postnatal day 8 and in adult (6–8-week-old) rats of both genders using immunohistochemistry. Our results show differential expression of P2X1 receptors in 9.4-14.2% of SGNs in the adult cochlea, and 14.2-23.3% in the cochlea of P8 pups. In most of these neurons, P2X1 receptors were co-expressed with peripherin-1, an established type II SGN marker. These results suggest a potential role for the P2X1 receptor as a modulator of auditory neurotransmission in type II spiral ganglion neurons.

## Introduction

The human cochlea, an approximately 1 cm organ deeply embedded in the temporal bone, is our peripheral organ for hearing. The human cochlea contains approximately 3400 inner hair cells (IHCs) and 14,000 outer hair cells (OHCs) across the 34 mm length of the basilar membrane [1], and 29,000–35,000 spiral ganglion neurons (SGN) [2]. During auditory transduction, sounds arriving at the cochlea as mechanical vibration depolarise IHCs to activate post-synaptic SGNs, which send axonal projections to the central auditory pathways in the brain. There are two broad classes of SGNs in the cochlea: type I and type II SGNs [3]. The cell bodies of both types of SGNs are located in Rosenthal’s canal in the central bony modiolus of the cochlea. Of the total SGN population, 90–95% are type I SGN. Type I SGNs are myelinated bipolar neurons with large cell bodies and axonal diameters, allowing rapid propagation of action potentials. These neurons are the primary auditory neurons that play a crucial role in transmitting sound information from the cochlea to the brain [4]. The dendrite from each Type I SGN forms synaptic connections primarily with only one IHC. Each IHC is innervated by 20 to 30 Type I SGNs. Functional observations of different thresholds of Type I SGNs, along with recent RNA-sequencing-based evidence, suggest that at least three subclasses of Type I exist in the mammalian cochlea [5–8]. On the other hand, type II SGNs make up only the remaining 5–10% of the total SGN population. Type II SGNs are pseudo-unipolar neurons, and their thin unmyelinated dendrites branch to innervate 5–30 OHCs, usually in the same row [9]. Their cell bodies are distributed throughout the spiral ganglion, with fewer found in the most apical region [10]. Type II SGNs have been suggested to be involved in sensing of cochlear trauma [9, 11].

P2X receptors are ATP-gated ion channels often localised in pre- or post-synaptic terminals, where ATP functions as a neurotransmitter [12]. Previous studies have described the expression of P2X1 receptors in the spiral limbus, Reisner’s membrane and SGNs in the cochlea of juvenile (P0-P6) and adult Wistar rats [13, 14]. Nikolic et al. (2001) demonstrated P2X1 immunoreactivity in both afferent and efferent neurite projections and the soma of SGNs in the developing rat cochlea with a developmental decline from P10 onwards [12]. Here, we report that the P2X1 receptor expression remains in SGNs after P10 in the Wistar rat cochlea. P2X1 immunoreactivity was observed in a subpopulation of SGNs likely representing Type II neurons, but was mostly absent from Type I neurons. These findings imply differential roles of P2X1 receptor signalling in the developing and mature rat cochlea.

## Method

Animal studies were approved by the Animal Ethics Committee at the University of Auckland (AEC approval No. 002251). Otherwise stated, all the chemicals were purchased from Thermo Fisher Scientific (Waltham, MA, USA). Wistar rats of both sexes were used in equal proportions in this study. P8 Wistar rat pups were decapitated using surgical scissors, and 6–8-week-old Wistar rats, considered young adults, were euthanised with an overdose of pentobarbital (90 mg/kg, ProVet NZ Pty Ltd, New Zealand) followed by cardiac perfusion with flushing solution (0.1M phosphate-buffered saline (PBS) with NaNO_2_) and 4% w/v paraformaldehyde. Temporal bones were extracted and immersed in 4% paraformaldehyde for 24 hours at room temperature. Cochleae from 6-week-old rats were decalcified by immersion in 4% w/v Ethylenediaminetetraacetic Acid in PBS at room temperature for two weeks, while this step was unnecessary for the P8 cochlea. For the organ of Corti (OoC), whole mounts and cryosections (at 30 μm thickness) were used for immunochemistry following our standard protocol [15], which includes incubation with blocking solution (2.5% v/v Triton X-100 in 0.1M PBS and 10% v/v normal goat serum or donkey serum for whole mounts, and 1% Triton X-100 v/v in 0.1M PBS and 10% v/v/normal goat serum/donkey serum for cryosections). All antibodies were diluted in antibody solution (0.25% Triton X-100 v/v in 0.1M PBS and 10% v/v normal goat serum/donkey serum for whole mounts, 0.1% Triton X-100 v/v in 0.1M PBS and 10% v/v normal goat serum/donkey serum for cryosections). The primary antibodies used were: rabbit polyclonal anti-P2X1 antibody (Alomone Labs, Jerusalem, Israel, Catalog# APR-001, 1:500 dilution), mouse anti-β III tubulin (IgG2a monoclonal, BioLegend, San Diego, CA, catalog No. 801213, 1:1000 dilution), goat polyclonal anti-peripherin-1 antibody (Everest Biotech, Upper Heyford, UK, catalog No. EB12405, 1:1000 dilution). For the peptide preabsorption control, the P2X1 receptor blocking peptide (Alomone Labs, Jerusalem, Israel, Catalogue # BLP-PR001) was mixed with the primary antibody in a 1 mg to 1 mg ratio and incubated for one hour at room temperature following the manufacturer’s protocol. Secondary antibodies used were: goat polyclonal anti-rabbit Alexa 594, goat polyclonal anti-mouse Alexa 488, donkey polyclonal anti-rabbit DyLight 594, donkey polyclonal anti-goat Dylight 488 (ThermoFisher, all used at 1:1000 dilutions). 4′,6-diamidino-2-phenylindole (DAPI, 300 nM final concentration) was added to the secondary antibody mixture. Between each incubation, tissues were washed with 0.1M PBS at room temperature four times (for 3 min, 5 min, 10 min and 20 min). The tissues were mounted with CitiFluorTM AF1 (Science Services, Germany) and imaged with confocal microscopy (Olympus FV1000, Tokyo, Japan). Z-stacks were obtained with a 20x objective lens and a 60x objective lens at 0.263μm/pixel and 0.24μm/pixel. Image analysis was carried out using FIJI (ImageJ). Total SGNs were counted based on anti-β III tubulin immunolabelling. These Z-stack images were imported into ImageJ software, and ImageJ ‘multipoint’ tool was used to label SGNs that were measured. SGNs were counted when a) a clear full-view of the nucleus (i.e. maximum diameter of the nucleus) was visible and b) the soma size surrounding the nucleus was approximately the largest for the particular SGN (i.e. optical section was taken at an approximately ~50% into the cell in Z-axis). These were to ensure that the same SGN would only be counted once within the Z-stack. This was further confirmed by re-assessing the entire Z-stack. The density of the total SGNs or total positive-P2X1 SGNs was calculated by dividing the total value by the volume of the Rosenthal canals of the Z-stack. The area of Rosenthal’s canal was estimated using the ‘free-hand’ tool to select the region of interest (RoI), and the area was measured for each slice in the z-stack. The SGN density was expressed as the cell count per 100 μm^3^ to give a number of cells/100μm^3^ as previously described [10] (Table 1). Within the z-stack, an average area was calculated, and then the area was converted into a volume by multiplying by the thickness of the Z-stack.

**Table 1:** Quantification of the population of SGNs stained with anti-P2X_1_ antibody

Density (SGNs/(100um)^3^)	Adult (*n* = 4)			P8 (*n* =4)		
	Apical	Middle	Basal	Apical	Middle	Basal
Strongly P2X_1_ positive number/(100μm)^3^	72 ± 30 (12.5%)	29 ± 7 (9.44%)	40 ± 9.0 (14.2%)	77 ± 2.5 (15.7%)	71 ± 3.0 (14.2%)	100 ± 24 (23.3%)
Total SGN number/(100μm)^3^	575 ± 180	307 ± 46	280 ± 120	490 ± 40	500 ± 34	430 ± 35

## Results

**Fig. 1:**
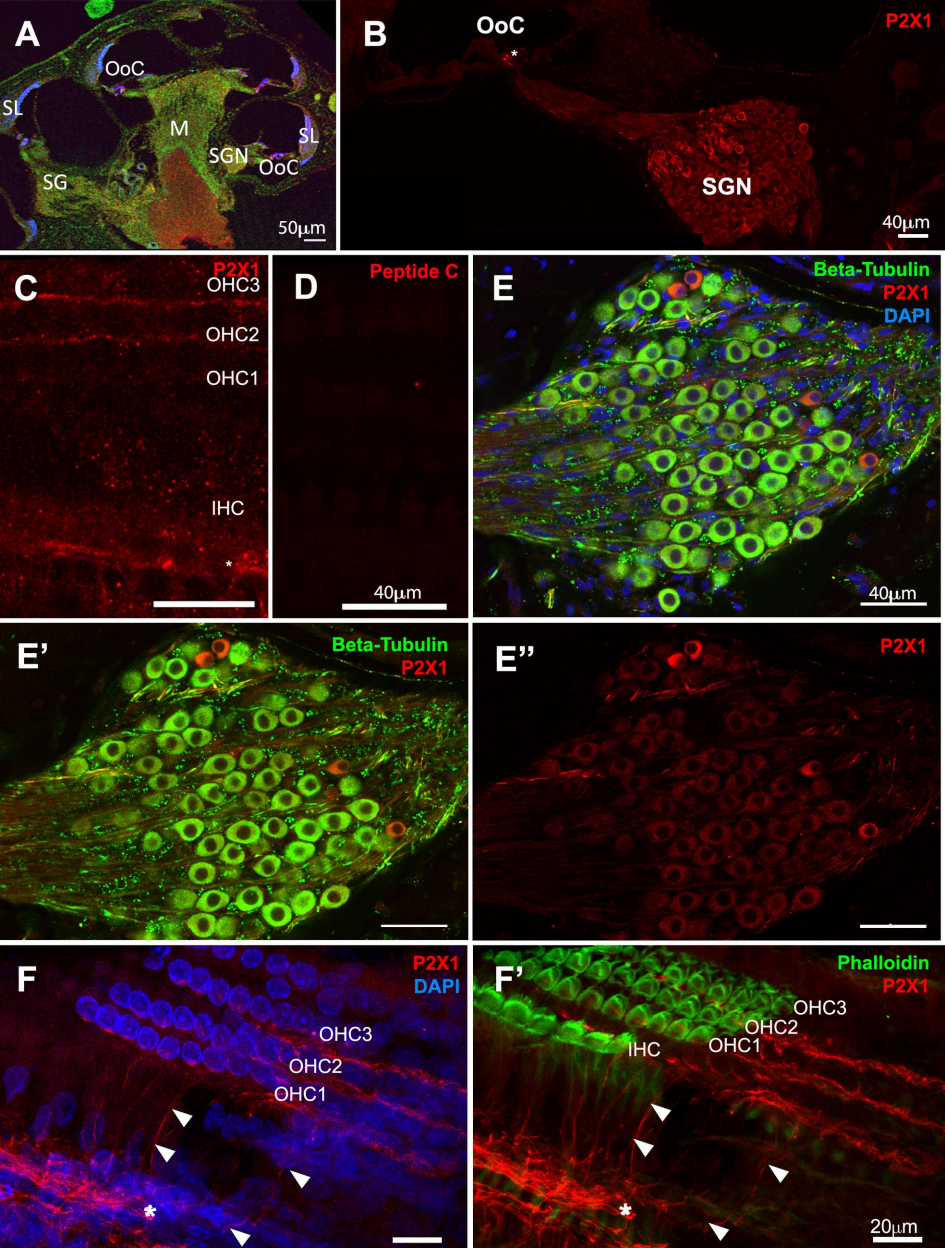
P2X1 receptor expression in the adult Wistar rat cochlea. Cryosections (**A**-**B**, **E**) and whole mount (**C**-**D**, **F**) from adult (6–8 week-old) Wistar rat cochleae were immunolabelled with anti-βIII tubulin (*green*) and anti-P2X1 antibody (*red*), and counterstained with DAPI (*blue*). (A-B) Representative image showing the overviews of rat cochlear cryosections (**A**) and the region with the organ of Corti and Rosenthal’s canal (**B**) showing P2X1 receptor expression in the organ of Corti (OoC) and the spiral ganglion neurons (SGN). (C-D) 6–8-week-old rat cochlea whole mount OoC preparation showing P2X1 immunolabelling, focusing on the SGN processes underneath the inner and outer hair cells (**C**) and the corresponding region in P2X1 antibody peptide block control (**D**). (**E**-E”) Rosenthal’s canal of cryosection from (**B**). (**F**-F’) 6–8-week-old OoC rat cochlea whole mount z-stack images showing tunnel crossing fibres labelled with anti-P2X1 antibody (arrowheads) and P2X1 immunolabelling at the base of the OoC (*). Scale bars, 50 *μ*m (A), 40 *μ*m (**B**-**E**), 20 *μ*m (**F**). Representative images from *n* = 4 cochleae from four different animals

**Figure 2.**
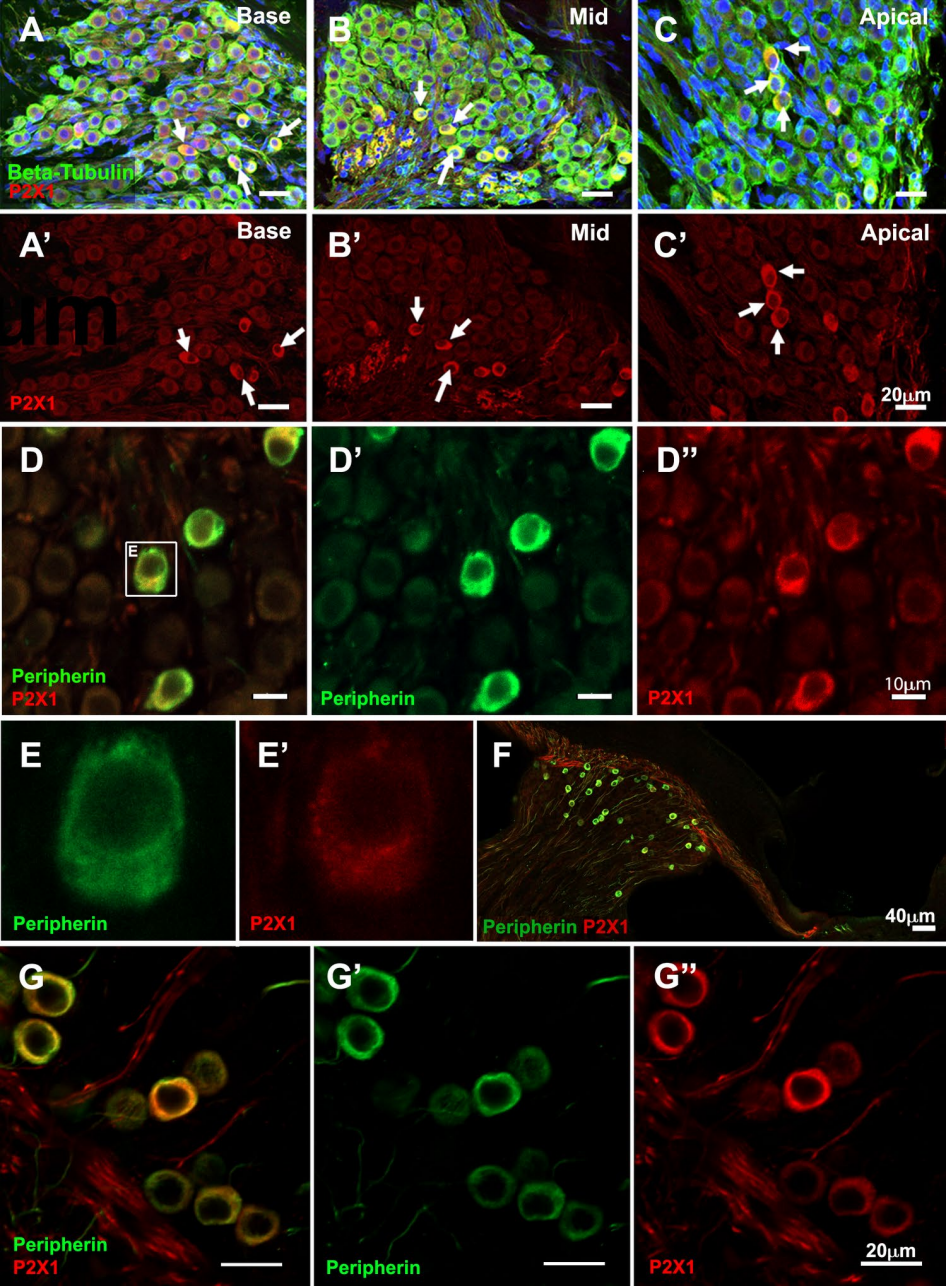
Co-expression of P2X1 receptors and peripherin-1 in Wistar rat cochlea. (**A**-**C**) Cryosections from adult (6–8 week-old) Wistar rat cochleae were immunolabelled with anti-βIII tubulin (green) and anti-P2X1 antibody (red), and counterstained with DAPI (blue). (**F**) White arrows indicate SGNs with strong P2X1 signals relative to other SGNs. Images were taken of the basal (**A**), mid-(**B**) and apical (**C**) regions of the cochlea. Scale bar = 20 μm for (**A**-**C**). (**D**-**G**) Cryosections from 6–8-week-old Wistar rat cochlea (**D**-**E**) and P8 cochlea (**F**-**G**) were co-labelled with anti-peripherin-1 antibody (*green*), and anti-P2X1 antibody (*red*). Images show the overview from the apical turn (**F**) and spiral ganglion neurons in the apical region (**G**-G”). Representative images from *n* = 4 cochleae from four different animals

The protein expression pattern of the P2X1 receptor was investigated in the cochleae of developing and adult Wistar rats using the commercially available anti-P2X1 antibody. The antibody was raised against the intracellular C-terminal domain of rat P2X1 (residues 383–399) and has been used in over 70 published articles, including testing in gene knockout tissues of the arterial smooth muscle [16] and male reproductive systems [17]. In the adult Wistar rat cochlea, a subpopulation of SGNs remained strongly immunolabelled with anti-P2X1 antibody (Fig. 1A, B). P2X1 immunolabeling was observed in the regions where SGN processes reached the OoC (Fig. 1B), which was attributed to SGN processes extending towards OHCs at higher resolution (Fig. 1C & F). Control tissues pre-absorbed with the corresponding peptide did not show immunolabelling (Fig. 1D). To visualise SGN, an anti-βIII tubulin antibody was used as a pan-SGN marker in the cochlea [18] (Fig. 1E, Fig. 2.A-C). Anti-βIII tubulin has been reported to be faint or absent in type II SGN in rats [19] and mice [20, 21]. This was observed in our study. P2X1 strong immuno labelling was observed in those cells weakly stained by anti-βIII tubulin antibody, which were sparsely distributed and tended to have smaller soma sizes than unlabelled SGNs (Fig. 1E, Fig. 2.A-C). A similar expression pattern was observed in P8 cochlea (data not shown). By labelling a whole mount preparation and examining Z-stacks counterstained with DAPI and phalloidin, the P2X1 immunolabeling was strong in the neural processes at the base of the OoC (Fig. 1F, *), extending over the tunnel of Corti (Fig. 1F & 1F’, arrowheads) towards OHCs (Fig. 1F).

P2X1 receptor expression in a subset of SGNs was observed in the apical, mid and basal turns of the cochlea (Fig. 2.A-C). The number of P2X1-positive SGNs was counted separately in apical, middle and basal turns of the adult cochlea and P8 cochlea to estimate the cell density (Table 1). More SGNs were observed in the apical turn (575 ±180 SGNs/(100μm)^3^) than in the basal turn (280±120 SGNs/(100μm)^3^) of the adult rat cochlea, consistent with the density pattern observed in cats [22]. The proportion of strongly P2X1-positive SGNs ranged between 9.4%−14.2% in the adult rat cochlea and between 14.2% - 23.3% in the P8 cochlea (Table 1). This is slightly higher than expected from the reported proportion of type II SGNs relative to the total number of SGNs [10]. We then tested if the subpopulation of P2X1-positive SGNs was type II SGNs by co-immunolabeling with the peripherin-1 antibody. Peripherin-1 is an intermediate filament protein selectively expressed by type II SGNs in the postnatal and adult cochlea [23, 24]. The anti-peripherin 1 antibody (Everest Biotech, # EB12405) has been validated in peripherin-1-knockout 129Sv/C57BL/6 mice [25, 26]. In the adult cochlea, SGNs labelled with anti-peripherin-1 antibody were also co-labelled with P2X1 receptors (Fig. 2.D). This was supported by quantitative analysis for most of the SGNs, though it should be noted that there were more peripherin-positive SGNs than P2X1-positive cells, indicating there are likely peripherin-positive, P2X1-negative neurons in a very small number (Table 2). At a higher resolution, both peripherin-1 and P2X1 receptors appeared to be co-expressed in the soma of type II SGNs, albeit with slightly different patterns; P2X1 receptors appeared more punctate closer to the nucleus, while peripherin-1 immunolabeling was distributed throughout the cytoplasm (Fig. 2.E). The high degree of overlap between P2X1 receptors and peripherin-1 immunolabelling was also observed in the P8 cochlea (Fig. 2.F, G, Table 2) with near-100% co-expression.

**Table 2:** Co-labelling of SGNs with anti-P2X1 antibody and anti-Peripherin-1 antibody

SGN Counts	Adult (*n* = 4)			P8 (*n* = 4)		
	Apical	Mid	Basal	Apical	Mid	Basal
Total number of SGNs (*n*=4 cochlea)	333	410	101	501	480	153
P2X_1_ positive	29	32	11	56	53	22
Peripherin-1 positive	45	41	1	68	53	24
Peripherin-1 & P2X1 positive	29	32	1	56	53	22

## Discussion

In the present study, P2X1 receptor protein expression in the adult and P8 Wistar rat spiral ganglion was mostly confined to type II SGNs, as evidenced by co-expression with peripherin-1. The number of peripherin-1 labelled or P2X1-labelled SGNs was slightly higher than expected; the proportion of the type II SGNs reported in the literature is approximately 5–10% in the mature mouse cochlea [10] and 7~8 % in rats [27]. The discrepancy may be due to sampling bias or strain difference. We also cannot eliminate the possibility that the P2X1 receptor is expressed in type II SGNs and a very small subset of type I SGNs. According to literature, the number of type II SGNs decreased from postnatal, pre-hearing onset to 3-week-old cochlea [10]. Our study showed a similar trend where the % of cells positively labelled for P2X1 was higher in the P8 cochlea than in the adult cochlea. Previously, studies have reported trends that type II SGNs are observed more frequently at the most lateral edge of Rosenthal’s canal [28–30]. Our observation of P2X1-labelled SGNs seems to align with these observed trends, further supporting that P2X1-labelled SGNs are type II SGNs. Near-100% co-expression of P2X1 and peripherin-1 likely demonstrates the utility of anti-P2X1 antibody as an alternative marker for type II SGNs in the mature cochlea.

Nikolic et al. (2001) demonstrated robust P2X1 immunolabeling in the majority of SGNs in the Wistar rat cochlea between E16 – P6. However, with further differentiation and maturation of the SGNs, P2X1 expression declines in type I SGNs but remains prominently expressed in type II SGNs in the mature cochlea. Cytoplasmic expression of P2X receptors is commonly observed [31]; P2X1 receptors in the soma of type II SGN may represent P2X1 receptors synthesized in the endoplasmic reticulum (ER) and in the process of being trafficked along neural processes.

Purinergic receptors (P2X, P2Y, and adenosine receptors) are broadly expressed in the cochlea [32] and the vestibular system [33]. SGNs express a broad range of purinergic receptor subtypes. All P2X receptors identified so far are expressed in the SGNs at the transcript level across different developmental stages, and some (e.g. P2X7 and P2X2 receptors) remain in more mature SGNs [32]. SGNs appear to express multiple P2X subtypes at varying transcript levels, with some evidence of their functional roles [11, 34–37]. Because P2X subtypes are capable of forming homo- and heteromeric channels in varying combinations [38], pharmacologically differentiating contributions from each P2X subtype is highly challenging. In SGNs, expression of other P2X receptors and some functions have been reported; for example, the developmental expression of P2X3 receptor plays a role in the refinement of SGN innervation [39]. A study based on knock-out mice suggests a role for P2X7 receptors in type II SGNs [40], although this finding contradicts with a previous study in rat cochlea, which showed expression of P2X7 receptors in glial cells and not SGNs [41].

Type II SGN axons make synaptic connections with postsynaptic interneurons in the anterior-ventral, posterior-ventral and dorsal cochlear nucleus [3, 42], then those to the medial olivocochlear neurons that send contralateral and ipsilateral medial-olivocochlear efferent back to OHCs in the cochlea to regulate OHC electromotility [42]. The knock-out of type II SGN processes reportedly abolished OHC suppression by medial-olivocochlear efferent [26]. However, this has been disputed by counter evidence that suggests the role of type II SGN serves a more modulatory function at the synapse level [43], and contributes to sensing trauma/damage in the cochlea [9], with a role to avoid further damage to the cochlea. In this regard, ATP signalling in type II SGNs has been postulated as the molecular mechanism of a ‘trauma detector’ in the cochlea, activated when OHCs are damaged [35, 44]. Liu et al. (2015) used Sprague-Dawley rat pups (P7-10) to demonstrate that an insult to OHC causes an ATP-dependent response in type II SGNs, which was abolished by a broad-spectrum purinergic blocker, pyridoxalphosphate-6-azophenyl-2', 4'-sulfonic acid (PPADS) [44]. The results from our study suggest a role for those P2X1 receptors in the type II SGN to be the detection mechanism of stress-induced ATP release. Further studies are required to confirm the functional expression of P2X1 receptors in the postsynaptic terminals of the type II SGNs at the OHC-Type II SGN synapses. In addition, it would be interesting to investigate P2X1 receptor expression at the type II SGN terminal in the cochlear nucleus and in the efferent innervation to the cochlea, to understand the ATP interplay with other neurotransmitters.

## Conclusion

This study demonstrated P2X1 receptor expression in type II SGN cell bodies and dendrites in adult (6–8-week-old) and juvenile (P8) Wistar rat cochlea. The specific localisation of the P2X1 receptors in type II SGN suggests a role in the modulation of efferent olivocochlear feedback to OHCs and a possible role as trauma detectors in OHCs.

## Data Availability

Data are available upon request to the corresponding author.
